# Lupus Cardiomyopathy and Nephritis Associated With Adalimumab and Cytomegalovirus Infection in a Patient With Seronegative Rheumatoid Arthritis: A Case of Rhupus Syndrome

**DOI:** 10.7759/cureus.34489

**Published:** 2023-02-01

**Authors:** Palapun Waitayangkoon, Lucas Chen, Jessica B Liu, Astrid Weins, Felipe Barbosa, Thomas Treadwell

**Affiliations:** 1 Internal Medicine, MetroWest Medical Center, Tufts University School of Medicine, Framingham, USA; 2 Pathology, Brigham and Women's Hospital, Harvard Medical School, Boston, USA; 3 Infectious Diseases, MetroWest Medical Center, Tufts University School of Medicine, Framingham, USA

**Keywords:** adalimumab, cytomegalovirus, lupus nephritis, sle and lupus nephritis, drug-induced lupus erythematosus, sytemic lupus erythematosus, cardiac lupus, tnf alpha, cytomegalovirus-cmv, adalimumab (humira)

## Abstract

Tumor necrosis factor-alpha (TNF-α) inhibitors are associated with lupus-like disease, known as anti-TNF-α-induced lupus (ATIL). Cytomegalovirus (CMV) was reported to exacerbate lupus in the literature. To date, systemic lupus erythematosus (SLE) triggered by adalimumab in the setting of CMV infection has never been described. We present an unusual case of a 38-year-old female with a past medical history of seronegative rheumatoid arthritis (SnRA) who developed SLE associated with the use of adalimumab and CMV infection. She had severe SLE features including lupus nephritis and cardiomyopathy. The medication was discontinued. She was initiated on pulse steroid therapy and discharged with an aggressive regimen for SLE, including prednisone, mycophenolate mofetil, and hydroxychloroquine. She remained on the medications until a year later upon follow-up. ATIL from adalimumab usually manifests only mild symptoms of SLE such as arthralgia, myalgia, and pleurisy. Nephritis is very rare, and cardiomyopathy is unprecedented. Concomitant CMV infection might contribute to disease severity. Patients with SnRA may have an increased risk of developing SLE later when exposed to such medications and infection.

## Introduction

Tumor necrosis factor-alpha (TNF-α) inhibitors are effective treatments for many autoimmune disorders such as rheumatoid arthritis (RA), ankylosing spondylitis, and inflammatory bowel disease [1]. The major concerns of these drugs are increased risk for infection, especially tuberculosis reactivation as well as immunogenicity elicitation, leading to undesirable adverse effects, including vasculitis, demyelination syndromes, glomerular interstitial nephritis, and anti-TNF-α-induced lupus (ATIL) [2]. ATIL is usually associated with mild symptoms such as skin and pleuropericardial abnormalities. We present a case of severe systemic lupus erythematosus (SLE) triggered by adalimumab (ADM) and transient cytomegalovirus (CMV) infection in a patient with underlying seronegative rheumatoid arthritis (SnRA).

## Case presentation

A 38-year-old biracial (mixed Black and Caucasian) female with a past medical history of SnRA presented with a two-week history of episodic fever, sore throat, productive cough, and bilateral lower extremity pain. She had immigrated from Brazil one month earlier. She has had SnRA and taken ADM every two weeks and daily sulfasalazine (SSZ) for two years. On arrival, she was tachycardic and febrile. Examination revealed scattered oral white lesions, conjunctival pallor, bibasilar crackles, and lower extremity muscle tenderness. There were no skin rashes or joint abnormalities. Initial complete blood count showed severe anemia, leukopenia with atypical lymphocytosis, and thrombocytopenia. Direct Coombs test was positive and ferritin, lactate dehydrogenase (LDH), and haptoglobin were elevated. Erythrocyte sedimentation rate (ESR) and C-reactive protein (CRP) were increased. A comprehensive metabolic panel showed elevated creatinine, aspartate aminotransferase (AST), alanine aminotransferase (ALT), and creatine kinase. Urinalysis was notable for proteinuria and hematuria without casts. The computed tomography (CT) of the chest, abdomen, and pelvis revealed diffuse adenopathy in the neck, supraclavicular, axillar, and iliac regions with bilateral pleural effusions and trace abdominal ascites. Extensive work-up for infections including COVID-19, HIV, tick-borne illnesses, syphilis, tuberculosis, malaria, leptospirosis, and hepatitis A, B, and C were negative except for the presence of both IgM and IgG antibodies to CMV. She received broad-spectrum antibiotics and a short course of ganciclovir. Antinuclear antibodies (ANA), anti-double stranded DNA (anti-dsDNA) antibodies, anti-histone antibodies, anti-Sjögren’s syndrome antigen A (anti-SSA/Ro), and IgG and IgM cardiolipin antibodies were positive with decreased C3 and C4 in the low normal range. Anti-Sjögren’s syndrome antigen B (anti-SSB/La), antinuclear ribonucleoprotein antibodies, anti-cyclic citrullinated peptide antibodies, and rheumatoid factor were negative (Table 1).

**Table 1:** Laboratory results on admission

Laboratory test	Value	Reference range
Hemoglobin	7.3 g/dL	12-16 g/dL
Total leukocyte count	1,500 cell/uL	4,000-11,000 cell/uL
Neutrophils	500 cell/uL	2,400-8,100 cell/uL
Platelets	73,000/uL	150,000-450,000/ul
Reticulocytes	0.2%	0.5-2.5%
Direct Coombs test	Positive	Negative
Erythrocyte sedimentation rate	>130 mm/1st h	0-20 mm/1st h
Urea	31 mg/dL	5-23 mg/dL
Creatinine	1.4 mg/dL	0.51-0.95 mg/dL
Aspartate aminotransferase	467 IU/L	10-34 IU/L
Alanine aminotransferase	155 IU/L	10-36 IU/L
C-reactive protein	6.1 mg/L	0-5 mg/L
Ferritin	9,231 ng/mL	13-150 ng/mL
Lactate dehydrogenase	1,667 U/L	135-235 U/L
Haptoglobin	<10 mg/dL	30-200 mg/dL
Creatine kinase	5,422 U/L	26-192 U/L
Urine protein creatinine ratio	2.9	<0.5
Antinuclear antibodies	Positive, homogenous, 1:1280	Negative
Anti-Ro/Sjögren’s syndrome antigen A (SSA)	Positive, >8 AI	0-0.9 AI
Anti-La/Sjögren’s syndrome antigen B (SSB)	Negative	Negative
Anti-double-stranded DNA	Positive, 1:640	<1:10
Anti-histone antibodies	Positive	Negative
Antinuclear ribonucleoprotein antibodies	Negative	Negative
Complement 3	32.2 mg/dL	90-180 mg/dL
Complement 4	10.7 mg/dL	10-40 mg/dL
Cardiolipin IgG Ab	25 GPL U/ml	0-14 GPL U/ml
Cardiolipin IgM Ab	49 MPL U/ml	0-12 MPL U/ml
Anti-cyclic citrullinated peptide antibodies	8 units	0-19 units
Rheumatoid factor	7 IU/mL	<14 IU/mL

**Figure 1:**
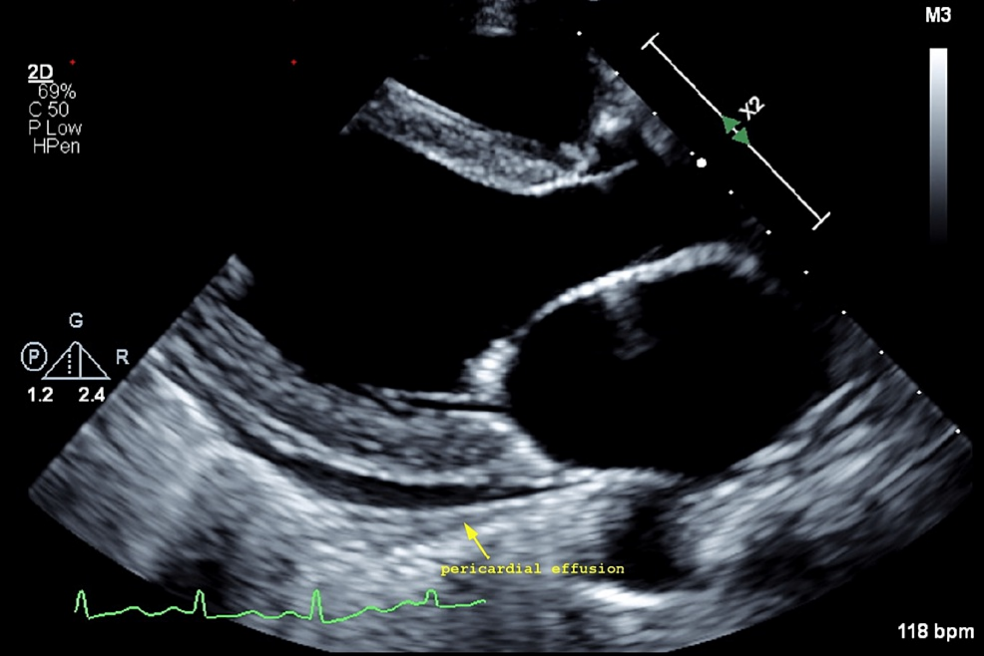
Transthoracic echocardiogram in the long-axis view demonstrating the presence of pericardial effusion indicated by the yellow arrow

**Figure 2:**
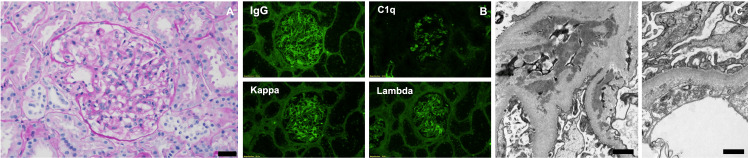
Kidney biopsy results (A) Periodic acid–Schiff-stained paraffin section: Glomerulus with mild mesangial hypercellularity and otherwise unremarkable architecture. Scale bar: 20 um. (B) Glomerular immune deposits with polyclonal reactivity; immunofluorescence staining for IgG, C1q, kappa, and lambda light chain is shown. (C) Extensive mesangial and focal subendothelial electron-dense deposits (left) and frequent tubuloreticular inclusions in endothelial cells (right; often seen in lupus nephritis). Podocytes show only segmental and mild foot process effacement. Scale bars: 1 um.

She was diagnosed with SLE and received pulse steroid therapy. She then developed hypoxia from vascular congestion. An echocardiogram showed severe global hypokinesis with severely reduced left ventricular systolic function (ejection fraction of 15-20%) and small pericardial effusion (Figure 1). Her autoimmune hemolytic anemia (AIHA) also worsened requiring two units of packed red blood cells. The kidney biopsy returned demonstrating mesangioproliferative lupus nephritis (class II), with frequent mesangial and more sporadic subendothelial and isolated subepithelial polyclonal immune deposits. Endocapillary hypercellularity or crescentic activity was not seen (Figure 2). She received prednisone, mycophenolate mofetil, hydroxychloroquine, metoprolol, lisinopril, furosemide, and colchicine. On a follow-up one year after ADM and SSZ discontinuation, she still required most of the medications on discharge including prednisone, mycophenolate mofetil, and hydroxychloroquine.

## Discussion

This patient presented with AIHA, leukopenia, thrombocytopenia, serositis with positive ANA, anti-dsDNA antibodies, histone antibodies, decreased C3, and positive anticardiolipin, consistent with SLE according to the American College of Rheumatology (ACR) 2019 criteria [3]. She was diagnosed with ATIL because she had taken ADM and her last dose was two weeks before her symptoms started [4]. It is interesting that she had severe features of SLE, severe AIHA, lupus nephritis, and cardiomyopathy, which are uncommon for ATIL described in the literature. Her symptoms of SLE continued to persist despite the cessation of the medications on a one-year follow-up. Moreover, she had evidence for acute CMV infection.

ADM is a TNF-α inhibitor that is commonly used to treat RA, psoriatic arthritis, and ankylosing spondylitis, as well as Crohn's disease and ulcerative colitis [1]. Common adverse effects are infections, which can sometimes lead to serious complications. Rarely, it can cause a lupus-like disease, known as ATIL. The incidence rate of ATIL from ADM was only 0.6% [5], which is far less than other TNF-α inhibitors, infliximab, and etanercept. In general, patients with ATIL have only minor symptoms of SLE such as arthralgia, myalgia, and pleurisy. Severe features are very rare. The prevalence of lupus nephritis was 7% in ATIL cases [2] and there were only a few case reports of nephropathy specifically in ADM users [6-8]. However, Ramos-Casals et al. described in a case series that cardiomyopathy has never been observed [9]. To our knowledge, our patient is the first case of ADM-associated lupus who had lupus nephritis and cardiomyopathy.

Based on the serology and polymerase chain reaction (PCR) results, this patient might have contracted a CMV infection prior to the presentation. This can explain some of her clinical features including pancytopenia with atypical lymphocytosis, diffuse lymphadenopathy, and elevated liver enzymes. The presence of CMV IgM and IgG antibodies but negative PCR is very suggestive; however, not diagnostic of acute CMV infection [10,11]. It is acceptable that CMV infection is associated with SLE exacerbation but its role as a trigger for the first SLE onset is debatable [12]. Several features of SLE and CMV infection frequently overlap, which makes it difficult to differentiate between them. Moreover, CMV IgM antibody may be seen in lupus as a non-specific autoantibody, hence the differentiation often relies on clinical features.

Interestingly, the patient also had severe AIHA with a low reticulocyte count. We believe the CMV infection may have caused bone marrow suppression thus contributing to pancytopenia. Reticulocytopenia accounts for 20% of the cases of infection-associated hemolytic anemia [13]. Alternatively, bone marrow involvement in SLE can lead to myelofibrosis, pure red cell aplasia, aplastic anemia, and myelodysplastic syndromes [14].

Transaminitis is one of the common laboratory abnormalities seen in patients with newly diagnosed SLE, as seen in our patient. It is important to keep in mind that other causes of liver diseases such as medications, viral hepatitis, and alcohol are more common and need to be excluded before concluding the diagnosis of lupus hepatitis [15]. Anti-ribosomal P antibody was recognized to be associated with lupus hepatitis, but the clinical usefulness is limited. In our case, it was difficult to determine if this was related to lupus itself, ADM, or CMV infection.

**Figure 3:**
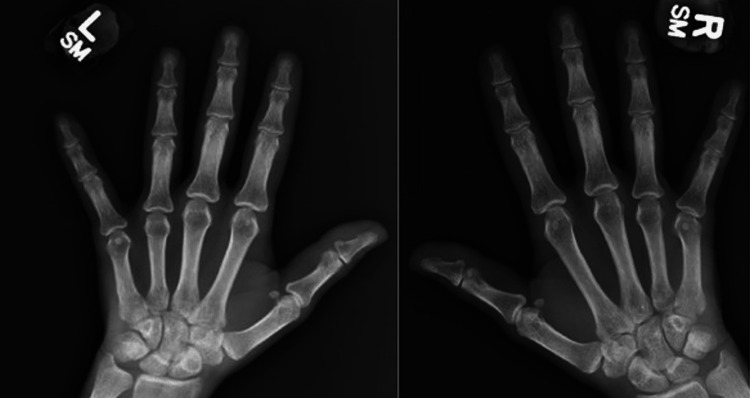
Radiography of the hands showing no evidence of bone erosions L: left; R: right.

Our challenge to ATIL diagnosis adds up to the patient’s previous diagnosis of SnRA. Without bone erosions on radiographs (Figure 3), it is possible that she was diagnosed with the condition by other features of RA such as a rheumatoid nodule or clinical response to disease-modifying antirheumatic drugs (DMARDs). This diagnosis is nonspecific and remains to be explored. In fact, the majority of SnRA patients meet the criteria for other rheumatologic conditions years after [16]. Although she has discontinued both ADM and SSZ for over a year, her regimen remained intensive similar to when she had on discharge. The lack of symptom resolution and severe major organ involvement might suggest a possibility of pre-existing SLE rather than underlying RA that was unmasked by ADM. Her overlapping features of RA and SLE satisfy the diagnosis of rhupus syndrome.

It could be argued that SSZ has caused drug-induced lupus in this patient. SSZ is a commonly used DMARD, which has been reported to cause ANA positivity without clinical relevance [17] and mild symptoms of SLE, especially rash and arthralgia [18]. Association with lupus glomerulonephritis has never been reported and it is rarely associated with anti-dsDNA [17]. For these reasons, we believe the diagnosis of ATIL is more suitable for this patient. Nevertheless, taking both medications together might contribute to the aggressive disease course, as seen in this patient.

The physiopathology of ATIL remains to be elucidated. It is believed that TNF-α inhibitors induce the development of lupus-related antibodies. Theories include the accumulation of nucleosomes from apoptotic cells inducing autoantibodies, medication-induced susceptible state to infection activating lymphocyte proliferation, a possible shift from Th1 to Th2 cytokine response upregulating antibody production [19], and molecular mimicry due to viral infection triggering the production of antibodies that cross-react with nuclear proteins [20]. Understanding the mechanism of ATIL may help prevent the condition or mitigate its severity in patients who take TNF-α inhibitors.

## Conclusions

ADM is less associated with lupus-like syndrome as opposed to other TNF-α inhibitors. Our case highlights the importance of long-term ADM use as a cause of severe lupus, which can manifest as nephritis and cardiomyopathy. Coexisting CMV infection might contribute to disease initiation or severity. Patients with SnRA may have an increased risk of developing rhupus later when exposed to such medications and infection.
